# Morphological Characterization and Quantification of the Mycelial Growth of the Brown-Rot Fungus *Postia placenta* for Modeling Purposes

**DOI:** 10.1371/journal.pone.0162469

**Published:** 2016-09-07

**Authors:** Huan Du, Pin Lv, Mehdi Ayouz, Arnaud Besserer, Patrick Perré

**Affiliations:** 1 LGPM, CentraleSupelec, Université Paris-Saclay, 92290, Châtenay-malabry, France; 2 ENSTIB/LERMAB, University of Lorraine, 88000, Epinal, France; USDA Forest Service, UNITED STATES

## Abstract

Continuous observation was performed using confocal laser scanning microscopy to visualize the three-dimensional microscopic growth of the brown-rot fungus, *Postia placenta*, for seventeen days. The morphological characterization of *Postia placenta* was quantitatively determined, including the tip extension rate, branch angle and branching length, (hyphal length between two adjacent branch sites). A voxel method has been developed to measure the growth of the biomass. Additionally, the tip extension rate distribution, the branch angle distribution and the branching length distribution, which quantified the hyphal growth characteristics, were evaluated. Statistical analysis revealed that the extension rate of tips was randomly distributed in space. The branch angle distribution did not change with the development of the colony, however, the branching length distribution did vary with the development of the colony. The experimental data will be incorporated into a lattice-based model simulating the growth of *Postia placenta*.

## Introduction

Wood is a traditional constructive material in many parts of the world due to its solidity, lightness and sustainability. Nowadays, wood and wood-based products, such as insulating panels, are commonly used to address the two main trends in building designs: the requirements for thermal performance and the use of renewable materials. Nevertheless, wood is easily bio-degraded by insects, fungi and bacteria, resulting in the damage of lumber structures and the reduction of building service life. Fungi are the primary causes of wood degradation, among which, the most destructive ones are brown rot fungi due to its rapid decaying mechanisms. Throughout Europe and North America, they are the most common wood decay fungi within buildings [1]. Recently, mathematical modeling has aroused great attention in the study of fungi [2, 3], which can forecast the behaviors of mycelial growth under different environmental conditions. Such modeling tools are likely to supplement costly and tedious experimental studies and are indeed absolutely required for service life to be accounted from the design stage. Our team is currently developing a model of fungal development based on the statistical reproduction of hyphal development. Our assumption is that the same mechanisms will be valid in wood and wood-based products, provided the porous geometry and the nutrients are accounted for via tropisms and/or modification of the growth statistics, such as elongation. Therefore, the first step of our modeling approach, which is the object of the present work, is to supply the model with statistics extracted from growth observations in free conditions (homogeneous medium and absence of resource limitations).

Different techniques have been used to visualize mycelial structure and to quantify its growth characteristics. A series of time-lapse 2D (bi-dimensional) images were recorded to visualize mycelial growth and structures by using photography, image scanner and conventional light microscopy [4–7]. However, they are obtained with low clarity and limited by sample thickness. Electron microscopy [8–10] has a large depth of field able to produce images free of focus blur, but samples need to be dehydrated, which results in inactivating living samples and prevents time-lapse observations. Alternatively, confocal laser scanning microscopy (CLSM) allows non-destructive optical sectioning of samples in observing living fungal cells and is used in the visualization of 3D (three-dimensional) structures of fungi at a high spatial resolution, which realizes the quantification of fungal surface area, volume and density allocation [11–18]. Moreover, in addition to the static visualization of fungi, dynamic observations of hyphal growth have been performed as well [19, 20].

*Postia placenta* is one of the most common brown rot fungi found in wood that is currently in service [21–23]. It decays wood at a rapid pace by degrading cellulose and hemicelluloses using self-produced enzymes and hydroxyl radicals, leaving lignin in place [24–27]. Apart from the biochemical aspect, the environmental factors, (e.g. temperature, moisture content, and oxygen depletion), that influence its growth rate in wood, have also been studied [28–30]. However, to our knowledge, there are no publications reporting the visualization of mycelial structures and quantitative measurement of growth characteristics of *Postia placenta* at the microscopic scale.

The objective of this work is to visualize *Postia placenta* and evaluate its morphological characteristics to produce a set of statistical functions able to feed a lattice-based model for simulating a free growth (without restriction of resources, like nutrients or growth space) of mycelium in homogeneous environment. Thus, the observations on the free-grown culture were performed in using CLSM during 17 days. 3D images and their 2D projection images were obtained in order to quantitatively measure and analyze the morphological and growth parameters of *Postia placenta*, such as tip growth rate, hyphal density, branch angle and branching location. Then, the mycelial growth in complex environments, such as porous morphologies and resource limitation, will be extended from the calibrated model. The simulation work based on the statistics derived in the present work will be the object of a full paper.

## Materials and Methods

### Materials

*Postia placenta* strain FPRL 280 and a media of malt extract agar (No.X923.2, Carl Roth) at a concentration of 33.6 g/L was used. Teflon culture dishes, with an internal height of 5 mm and inner-radius of 7.5 mm, covered by glass coverslips with a thickness of 0.14 mm were designed for the observations using confocal microscopy. Vacuum grease (Carl Roth), which is non-toxic to fungi, was applied at regular points along the circumference of culture dishes to fix coverslips.

### Methods

#### Inoculation and culture

750 *μL* malt extract agar was added into the culture dish to obtain a ∼4.2 mm-thick media. To avoid the contact of the suspension with the coverslip and to reduce the amount of small hyphal fragments in the inoculation, 5 *μL* of the suspension, (*Postia placenta* strain FPRL 280 mixed in sterilized water), was inoculated on the media. Several drops of the vacuum grease were put on the edge of the culture dish to fix the coverslip, which allowed sufficient oxygen supplies and, simultaneously, reduced the risk of contamination. Then the culture dish was kept in an incubator at 20°*C* for 72 hours before observations. Another ten cultures were prepared as controls at the same time and maintained at 20°*C* for one month. The mycelial growth in 2D dimension began to be restricted by the surface of the culture dish after two-week growth. Then, the colonies regained rapid growth towards z direction thereafter till one month. This proves that the nutrients in media were sufficient at least for one-month growth of *Postia placenta*.

#### Fluorescence staining

Calcofluor White M2R (Fluorescent Brightener 28, Sigma Aldrich), which stains fungal cell walls, was prepared as a 0.1%(*w*/*v*) stock solution in distilled water. In each staining, the stock solution was diluted to 0.001%(*w*/*v*) and was added into the culture by syringe filter (0.2*μm*). After 5 minutes, the dye was rinsed away gradually with sterilized water. The low concentration of the dye and short time of staining allowed for normal growth of hyphae [31] and, simultaneously, few residues of the fluorescent dye remaining in the media. The staining was repeated every 4 or 5 days to ensure a high signal-to-noise ratio while keeping low laser power, because the fluorescence intensity fades when exposed to the wavelength of 405*nm* (the selected laser line).

#### Confocal observation

Observations were performed at room temperature (∼20°*C*) during 17 days using a Zeiss LSM 700 Laser Scanning Confocal Microscope (CLSM). The UV diode laser, at a wavelength of 405*nm*, was used for illumination. Fluorescence of Calcofluor White M2R was captured through a band pass filter at a wavelength of 420 − 475*nm*. The sample was evaluated every 2–3 days. A Plan-Apochromat 10×/0.45 M27 objective lens was used to obtain the image, and each image was averaged across 4 scans. Since the medium was transparent and the dyes transferred to the penetrative hyphae, the whole colony could be visualized, including the surface and penetrative hyphae, (S1(a) Fig). As the mycelium developed, the sizes of digital images in the xy plane increased from approximately 4000*μm* × 4000*μm* to 9000*μm* × 9000*μm*. Pixel size in the xy plane was 1.25*μm*×1.25*μm* to be able to visualize the individual hyphae, as the hyphal diameter was greater than 2*μm*. The depth on the z-axis was approximately 600*μm* and the interval distance between every two adjacent xy slices was approximately 20*μm*. A 3D image was obtained through reconstructing the successive xy slices in each observation (partly shown in S1(b) Fig). A 2D projection image was obtained as well for each observation using the method of maximum intensity projection (MIP) [32], which consists of projecting the voxel with maximum intensity on every view throughout the volume onto a 2D image (S2 Fig). By using MIP of the color-coded slices, color projections were also generated to distinguish the hyphae at different depths.

#### Measurement and analysis

Optical slices in each 3D image were converted to binary masks using the triangle threshold method. The background noise caused by residual dyes was reduced by a selective median filter in ImageJ (NIH, Bethesda MD). In each 3D image, the number of voxels that contain hyphae in all slices were counted and plotted as a function of time to illustrate the evolution of biomass growth.

Tip extension length, (Δ*l*_*tip*_), was measured and obtained by comparing the same hypha in the overlapped two 2D projection images of consecutive observations using ImageJ (S3 Fig). The tips were classified into two categories: active tips (Δ*l*_*tip*_ > 0) and dormant tips (Δ*l*_*tip*_ = 0, defined as temporal non-extending tips which can regain the activity). The hyphae that could be visually discriminated were measured. The extension rate for each tip, as well as the average extension rate of the active tips on each image, was calculated using Eqs (1) and (2):

$$R_{tip}\left(i;x,y\right)=\frac{\Delta l_{tip}\left(i;x,y\right)}{\Delta t_{tip}}$$ (1)

$$\langle R_{tip}\rangle =\frac{1}{N_{atip}}\sum_{i=1}^{N_{atip}}R_{tip}\left(i\right)$$ (2)

where *R*_*tip*_(*i*; *x*, *y*) (*μm*/*h*) is the extension rate of tip *i* and the coordinates (*x*, *y*) are its position. Δ*l*_*tip*_(*i*; *x*, *y*) denotes the extension length of tip *i* when it reaches (*x*, *y*) in the interval of time Δ*t*_*tip*_. 〈*R*_*tip*_〉 is the average tip extension rate and *N*_*atip*_ is the number of active tips. The distribution of tip extension rate for each image was calculated by Eq (3):

$$\begin{array}{c} f\left(x_{i}\right)=\frac{n_{i}}{n_{t}\Delta x} \end{array}$$ (3)

where *x* is the data to be classified (in this case the tip extension rate, *R*_*tip*_, was the sample data); *n*_*i*_ the number of data in the *i*^*th*^ interval [*x*_*i*_ − Δ*x*/2, *x*_*i*_ + Δ*x*/2); *n*_*t*_ the sample size and Δ*x* the width of the interval; *f*(*x*_*i*_) the probability density of x falling within the *i*^*th*^ interval. Moreover, the quantities of new emerged branches per hypha, as well as, the ratio of active tips from the measured tips were quantified for each image.

**Fig 1:**
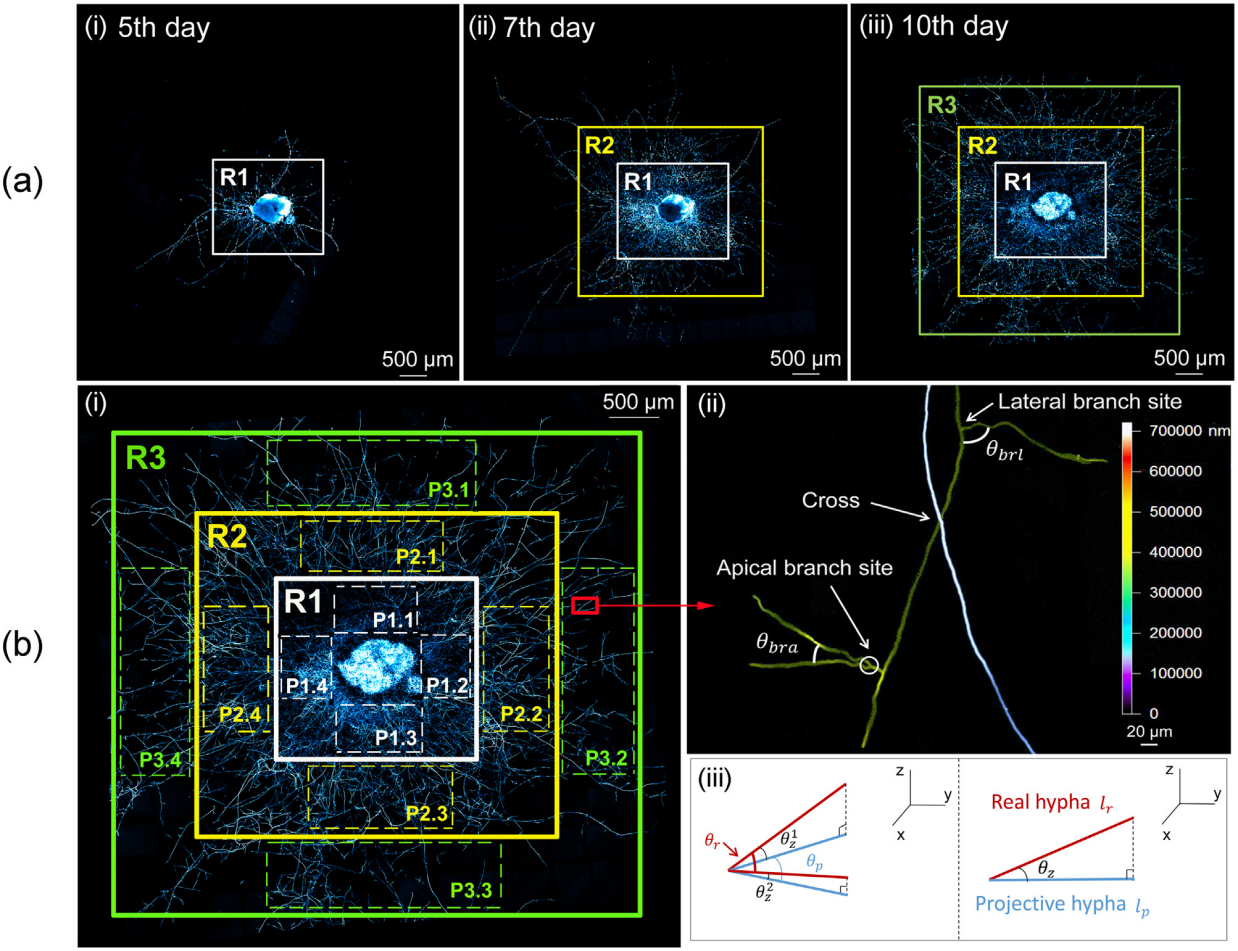
Method of the measurement of branching characteristics. (a) Three regions with equal gap to partition the young and old hyphae: (i) R1, (ii) R2 and (iii) R3, covering almost the entire colony on the 5th, 7th and 10th day following inoculation. (b) (i) Four portions selected for the measurement in each of the three regions (e.g., P1.1, P1.2, P1.3 and P1.4 in R1). (ii) Zoom of a color projection for measurement to show a cross of two hyphae with two types of branch sites and branch angles (AB angle (*θ*_*bra*_) and LB angle (*θ*_*brl*_)). The color bar shows the depth of the mycelium along the z-axis. (iii) Errors between real values and projective ones caused by z-direction growth of hyphae: (left) real branch angle (*θ*_*r*_), projective branch angle (*θ*_*p*_), and angle of the real hypha to the xy plan (*θ*_*z*_); (right) real branch length (*l*_*r*_) and projective branch length (*l*_*p*_).

Due to the linear and radial growth of hyphae, the biomass of mycelium depends on the total length of hyphae not the area of colony. Thus, three regions, (R1, R2 and R3), with equal gap were successively divided covering almost the entire colony on the 5th, 7th and 10th day following inoculation (Fig 1(a)). The selected regions were rectangular, which was more suitable for image processing but not ideal for measurement because of the radial growth of the colony. So four portions were selected in each region for quantitative comparison (Fig 1(b)-(i)), which allowed that the areas analyzed for each region were proportional to the radius. The shape and position of each portion chosen in the same region was representative of an “average” distance to the inoculum. In 3D images, the hyphal voxels of every four portions in each region were counted and normalized by the total number of voxels of all portions in the final observation. The data of each region was then plotted as a function of time. Curves from R2 and R3 were translated along the time axis to coincide with that of R1. The age of R1 was defined as the duration of the development of the colony since the inoculation, while the age of R2 and R3 was obtained by an age gap through this translation.

There are two types of hyphal branches, the apical branch (AB) and the lateral branch (LB), each distinguished by their formation patterns [33] (Fig 1(b)-(ii)). The AB emerges from a hyphal tip and two daughter branches develop symmetrically around this tip. Moreover, the LB is a new branch formed from a site distal to the hyphal tip. Accordingly, there are two types of branch angles and branch sites based on these types of branches. An AB angle is formed by two ABs and a LB angle is defined by the LB and the growth direction of the mother hypha. The location of branch development is denoted as one branch site, differing from a cross that is the intersection of two hyphae at different depths (Fig 1(b)-(ii)). Moreover, the branching length is defined as the hyphal length between the two adjacent branch sites. Next, the two types of branch angles, the number of AB sites and LB sites and the branching length were quantified and measured in each region from color projection images using the proprietary Zeiss software (ZEN 2012, black edition). The distributions of two branch angles, as well as, branching lengths were calculated using Eq (3) where the measured branch angle *θ* and branching length *l* were the sample data. Due to the projection in 2D of the real 3D structure, an error is possible for some of the measured branch angles and branching lengths. The projection error due to the z-component of the hyphal direction is shown in Fig 1(b)-(iii). This error was calculated using Eqs (4) and (5):

$$\epsilon_{\theta }=\theta_{r}-\theta_{p}=arccos\left(sin\theta_{z}^{1}sin\theta_{z}^{2}cos\theta_{p}+cos\theta_{z}^{1}cos\theta_{z}^{2}\right)-\theta_{p}$$ (4)

$$\epsilon_{l}=\varphi_{l}\times l_{p}=\left(1/cos\theta_{z}-1\right)\times l_{p}$$ (5)

where *ϵ*_*θ*_ is error of angle; *θ*_*r*_ real branch angle; *θ*_*p*_ projective branch angle; *θ*_*z*_ angle between real hypha and the xy plan, calculated by *arctan*(Δ*z*/Δ*l*); *ϵ*_*l*_ error of length; *φ*_*l*_ error coefficient, which was defined as the ratio of the error of length to the projective length; *l*_*p*_ projective branching length. Due to the color projection, the distance of z-direction growth (Δ*z*) and the corresponding projective distance (Δ*l*) can be measured and used to estimate *θ*_*z*_.

## Results and Discussion

3D images were reconstructed by the confocal slices, which allowed to calculate the growth of the biomass. 2D projection images were also obtained to observe the mycelial growth and to measure its morphological characterization. As shown in S2 Fig, the *Postia placenta* grew from a central inoculum block and 8 instants were recorded on the 3rd, 4th, 5th, 7th, 10th, 12th, 14th and 17th day, following inoculation. During the initial 5 days, the hyphal elongation was dominant, while on the 7th day, a visible emergence of branches occurred. Next, a symmetric colony formed and enlarged its area by tip extension, as well as branching. After the 14th day, the colony nearly reached the edge of the culture dish and could not continue to increase its area. Overall, the colony remained radially symmetric as it developed over time. Additionally, the mycelial surface density increased over time at a given location but with a distinct spatial gradient as the density decreases with the colony radius. This phenomenon is consistent with the fractal nature of mycelial growth [34, 35].

**Fig 2:**
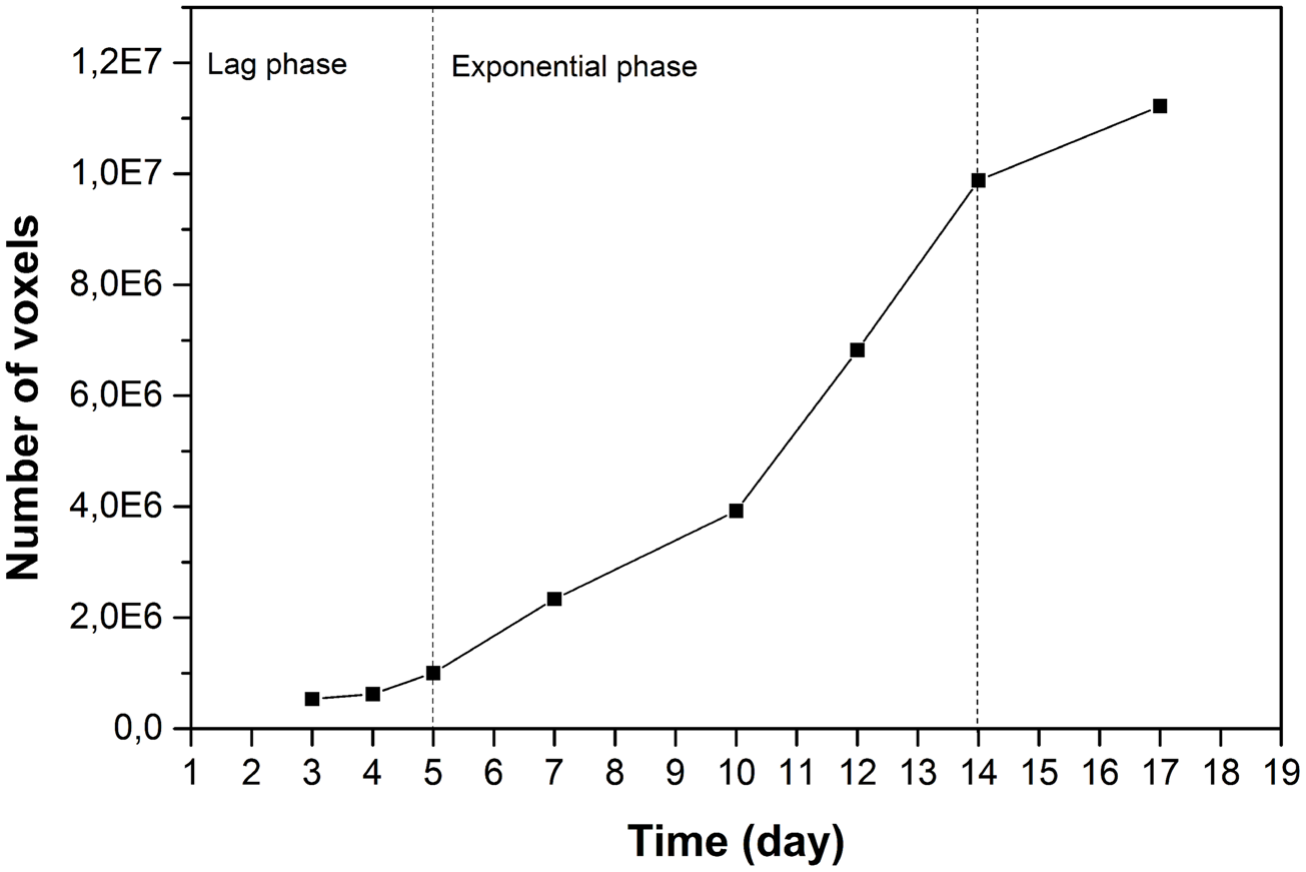
Biomass growth of the whole colony as a function of days post inoculation.

Biomass growth is a global index used to represent the status of mycelial growth. In Fig 2, the biomass growth is presented as a function of days post inoculation. The curve exhibits a lag phase of 5 days, during which the biomass growth was not considerable. The mycelium may be adapting and exploring the new environment and inducing its transport systems. The exponential phase began on the 5th day, when a rapid multiplication of hyphae occurred. As hyphal branches were initiated, the hyphae extended to the uncolonized regions and the increase of biomass exponentially increased. A stationary phase occurred after the 14th day, as evidenced by a gradual decrease of growth, potentially due to the limited planar space.

The morphological characterization of mycelia, including the tip extension rate and the branching parameters, was obtained by image processing and subsequently analyzed. The number of measured tips (*N*_*tip*_) in each overlapped image is shown in Table 1. The average proportion of active tips (*k*_*active*_) over all measurements was estimated as 0.72 and 0.70 during the lag and the exponential phase. And during the exponential phase, this proportion was high (0.77) from the 5th to the 12th day, and then decreased to 0.5 because the hyphal density became very high and a part of tips approached the edge of the culture dish. The average extension rate of the active tips (〈*R*_*tip*_〉) between two consecutive observations are also listed in Table 1 together with the new emerged branch number per hypha (*φ*_*br*_), which is the increment of branch number (Δ*N*_*br*_) over the number of tips (*N*_*tip*_). *φ*_*br*_ arose rapidly from 0.3 to 2.6 on the 7th day, then decreased to 1.8 and remained at this level for 7 days. The ratio (*k*_*lag*_) of the average value of *φ*_*br*_ during the lag phase to that during the exponential phase is 1/5. Thus, although the average proportion of active tips was slightly higher in the lag phase, the increase of biomass was not so high just because there were much fewer new tips (generated by emerged branches) in the lag phase than in the exponential phase. The peak of the the average tip extension rate coincides with that of new emerged branch number per hypha (*φ*_*br*_). It increased rapidly between the 5th and 7th day from 16.6 *μm*/*h* to 28.8 *μm*/*h*, then decreased to around 14 *μm*/*h* until the 14th day. From the 14th day, it decreased much lower. As a result, the tip extension rate and the branching emergence rate are both tied to the mycelial growth phase. Throughout the lag phase, tip extension dominated and few branches emerged, as also reported by J. Meletiadis *et al.*, 2001 [36]. Following the lag phase, both activities contributed to the rapid multiplication of day 7. Then, the average tip extension rate and the branching emergence rate remained nearly constant, which resulted in the continuance of the exponential phase until the 14th day.

**Table 1:** Average tip extension rate obtained using Eq (2) and relative increase of branch number per hypha during two consecutive observations.

Days after inoculation	3	4	5	7	10	12	14	17
**Number of measured tips** (*N*_*tip*_)	–	86	119	132	144	150	190	224
**New emerged branch number per hypha** *φ*_*br*_	–	0.3	0.3	2.6	1.8	1.4	1.4	0.9
**Average extension rate of active tips** 〈*R*_*tip*_〉 (*μ*m/h)	–	11.4	16.6	28.8	15.5	12.7	13.9	5.3

**Fig 3:**
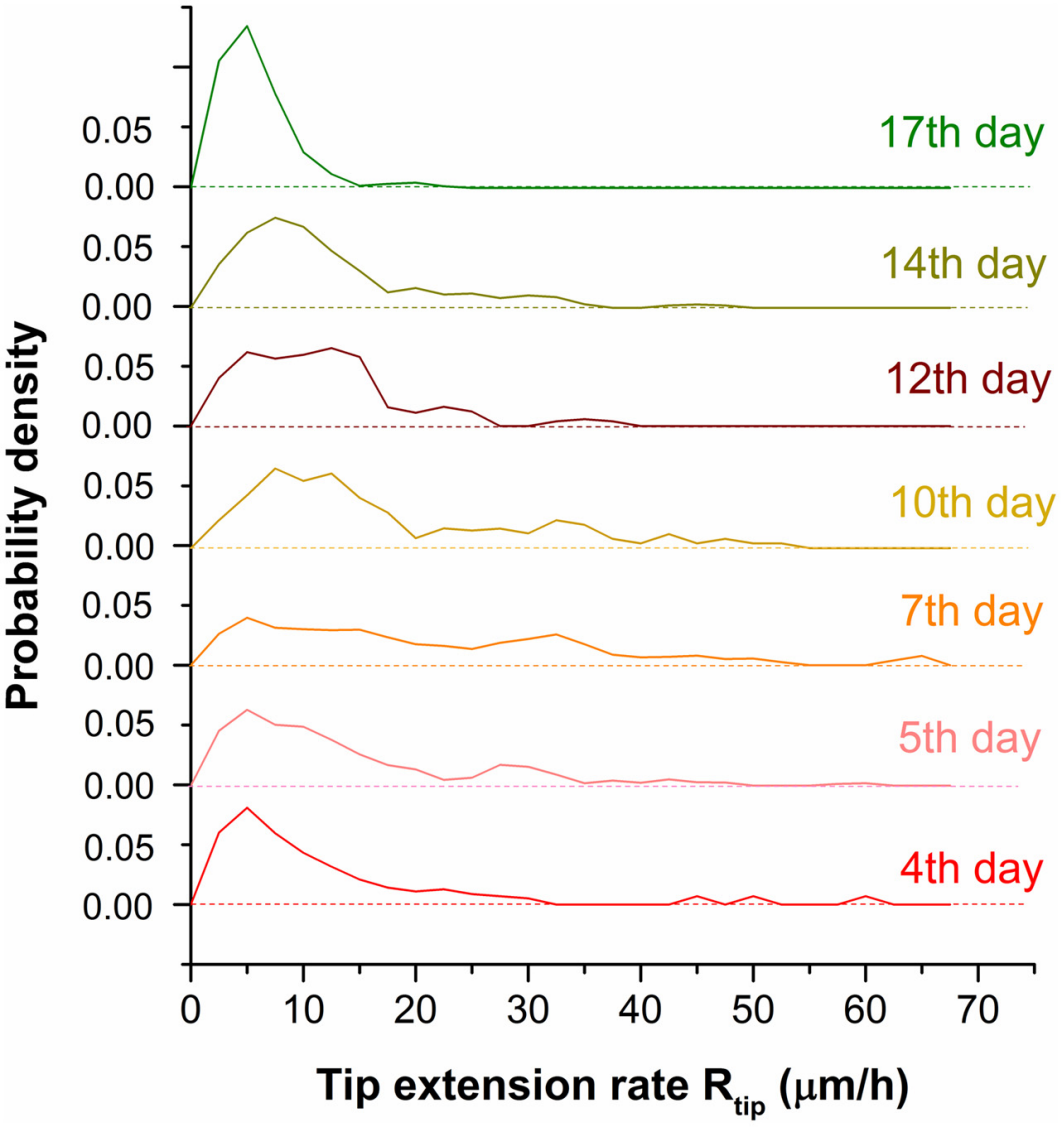
Distribution of the extension rate (*R*_*tip*_(*i*; *x*, *y*)) of active tips calculated during two consecutive observations. Different observations are displayed from the 4th to 17th day post inoculation.

**Fig 4:**
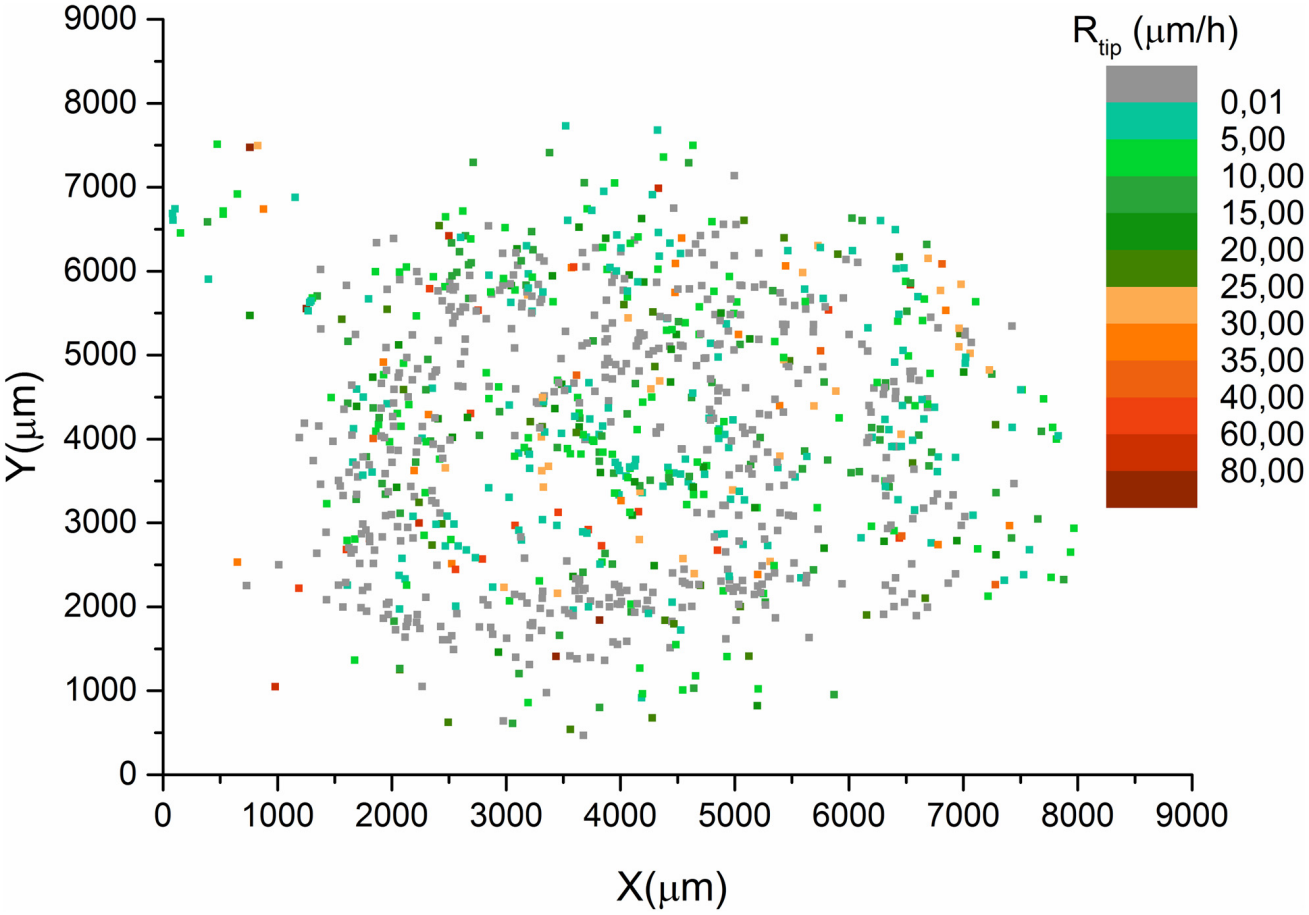
Spatial distribution of tip extension rate (*R*_*tip*_(*i*; *x*, *y*)) during 17 days of observations. (*x*, *y*) are the coordinates of the position of tip *i*. The colors display the different ranges of rate: from green, less than 25 *μm*/*h*, to orange for the > 25 *μm*/*h*. Grey points represent dormant tips.

Although the average tip extension, (calculated by Eq (2)), provides a global view, it cannot absolutely represent the tip extension rate because of the important deviation of individual values from the average. Indeed, individual values are spread over a wide range as shown by the distributions, calculated by Eq (3), in Fig 3. Fig 4 depicts the spatial distribution of tip extension rate calculated by Eq (1) during the 17 days. The green points represent the rates smaller than 25 *μm*/*h*, while the orange ones represent the higher rates. The green and orange points are randomly distributed over the entire domain, which indicates that the tip extension rate is randomly distributed in space. Therefore, the formation of the colony generally presents a radial symmetry over time, despite the irregular form of the inoculum. The fact that the extension rate of the same tip varies randomly with time has also been reported by Sampson K., 2003 [37].

**Fig 5:**
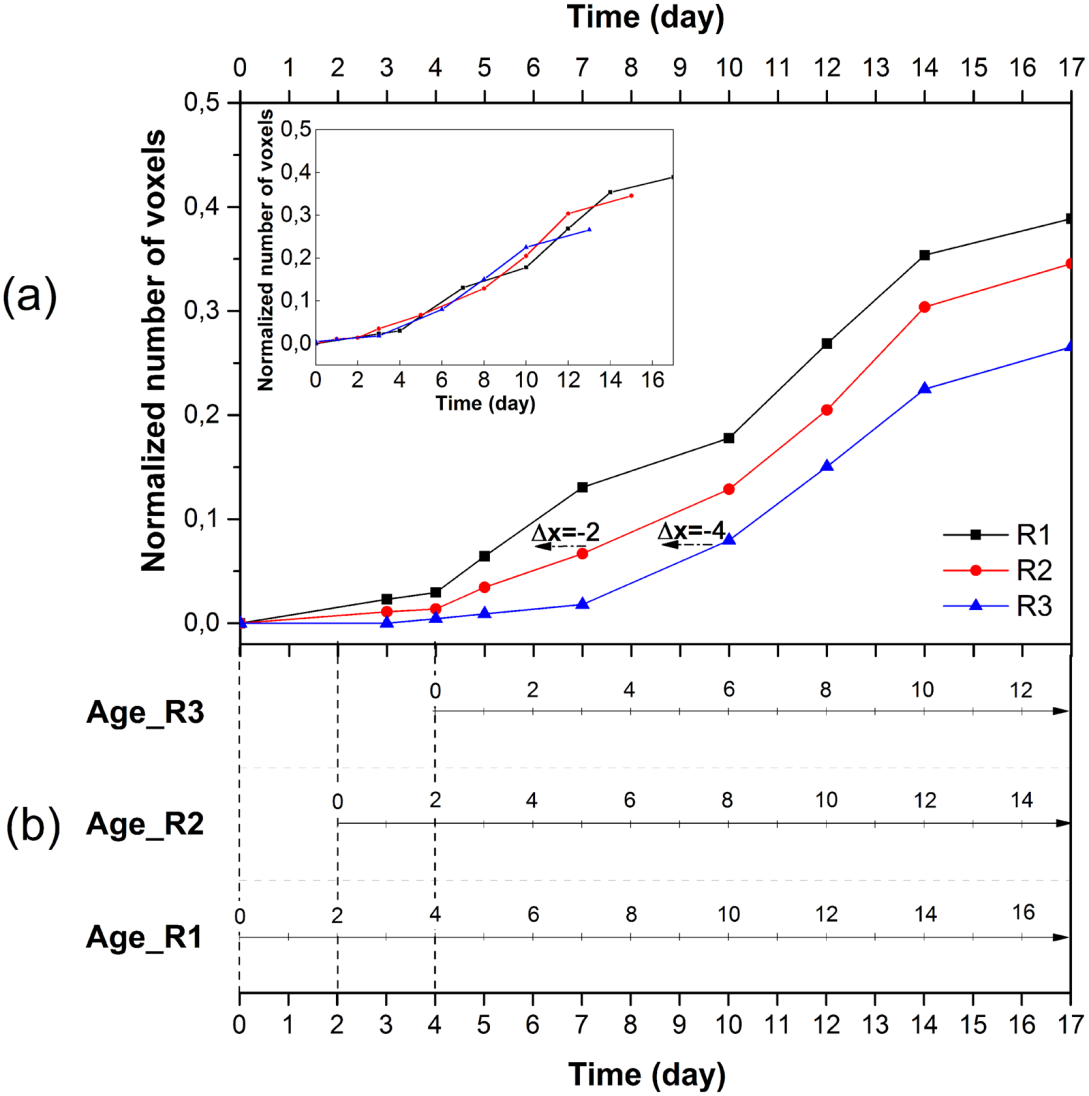
Age of region for R1, R2 and R3. (a) Biomass growth measured throughout the 4 portions of R1, R2 and R3 as a function of days after inoculation (the inset graph shows the three curves translated by Δ*x*). (b) The starting time of growth for R1, R2 and R3 is the inoculation day, the second day and the fourth day. The age axis is related with time axis to determine the age of each region on different observation days (E.g. the age of R1 on the 12th day is 12 days old but 10 and 8 days old for R2 and R3; the R1, R2 and R3 are 10 days old respectively after 10 days, 12 days and 14 days.) The age of region represented approximately the age of the oldest hyphae in that region.

As shown in Fig 5(a), the biomass growth in each region displayed a similar trend. The curves of R2 and R3 were translated by -2 days and -4 days along the time axis, respectively. Following translation, the three curves are nearly superimposed, which reveals that the biomass behavior in the three regions is similar (the inset graph in Fig 5(a)). Fig 5(b) illustrates the age of each region. The estimated starting time of hyphal growth in the three regions was 0, 2 and 4 days following inoculation. Thus, Fig 5(b) serves as an age axis for Fig 5(a).

**Fig 6:**
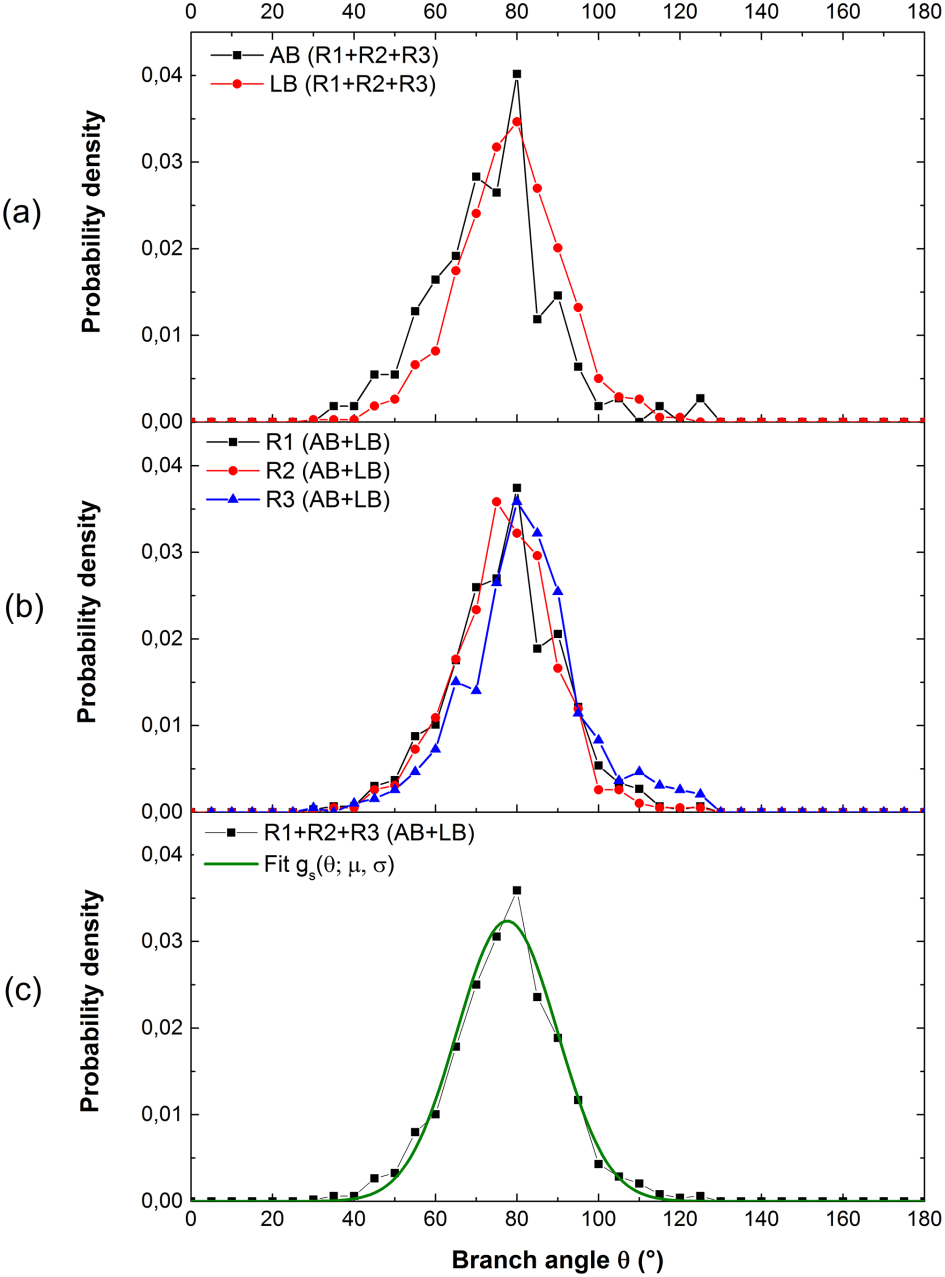
Distribution of branch angle measured in the observation on the 12th day. (a) AB angles and LB angles in three regions; (b) two types of branch angles respectively in R1, R2 and R3; (c) two types of branch angles in three regions and fitted curve. The similar branch angle distributions were observed in each of the different regions and they follow a Gaussian distribution.

An additional important branching parameter to simulate the hyphal morphology is the branch angle. AB and LB angles were measured in the four portions of R1, R2 and R3 in the 12th-day observation. The population of the angle sample is approximately 1200, including ∼400, ∼500 and ∼300 data measured respectively in R1, R2 and R3, among which there were ∼960 LB angles and ∼240 AB angles. A comparative plot of the AB angle distribution and the LB angle distribution in all three regions (Fig 6(a)) reveals that the type of branches has negligible effect on branch angle distribution. Fig 6(b) exhibits a similar branch angle distribution in R1, R2 and R3. According to Fig 5(b), these three regions on the 12th day were 12, 10 and 8 days old, which indicates there are no visible differences in the branch angle distribution at different ages of region. Alternatively, it is not influenced by different hyphal densities or by the age of the mother hypha from which the new branches emerged. Overall, the branch angle distribution of *Postia placenta* is not affected by the age of region, or by the branch type. The branch angle remained approximately 80°, close to the right angle, which appeared to maximize the area covered by the colony. The error of the branch angle between real value and projective value caused by the z-direction growth of hyphae calculated by Eq (4) was shown in S4(a) Fig. Considering around 200 errors out of 1200 angles in total, there are only 10 absolute errors greater than 5°, (0.8% of 1200 measured data and the relative error is 6.3% by the average of angles—80°), and 46 ones greater than 2°, (3.8% of 1200 measured data and the relative error is 2.5% by the average of angles—80°). As a result, we can assume that the error of the angle did not impact the branch angle distribution. The curve fitting was then implemented to the probability density of all measured angles (AB and LB angles in all three regions) using an evolutionary algorithm to minimize the residual sum of squares (RSS) (Eq (6)) between the experimental data and the Gaussian distribution. RSS is defined as:

$$RSS=\sum_{i}{\left(f\left(x_{i}\right)-g\left(x_{i}\right)\right)}^{2}$$ (6)

where *x*_*i*_ is the *i*^*th*^ explanatory variable, *f*(*x*_*i*_) is the *i*^*th*^ value of the probability density calculated in experiment and *g*(*x*_*i*_) is that of the fit. Normalized Gaussian function reads as follows:

$$g_{s}\left(\theta ;\mu ,\sigma \right)=\frac{1}{\sigma \sqrt{2\pi }}exp\left(-\frac{{\left(\theta -\mu \right)}^{2}}{2\sigma^{2}}\right)$$ (7)

where *θ* represents the branch angle, *μ* = 77.6° the mean of the distribution and *σ* = 12.3° the standard deviation. According to the fit, more than 68% of the branch angles were within the interval: *θ* ∈ [65°, 90°]. The accuracy of the fit was measured by RSS, which characterized the global quality of the fit. Fig 6(c) illustrates a low RSS of 5 × 10^−5^, which represents strong agreement between the experimental data and the fit. Eq (7) with the identified parameters will be applied in the modeling of mycelial growth to determine the formation of AB and LB angles.

**Fig 7:**
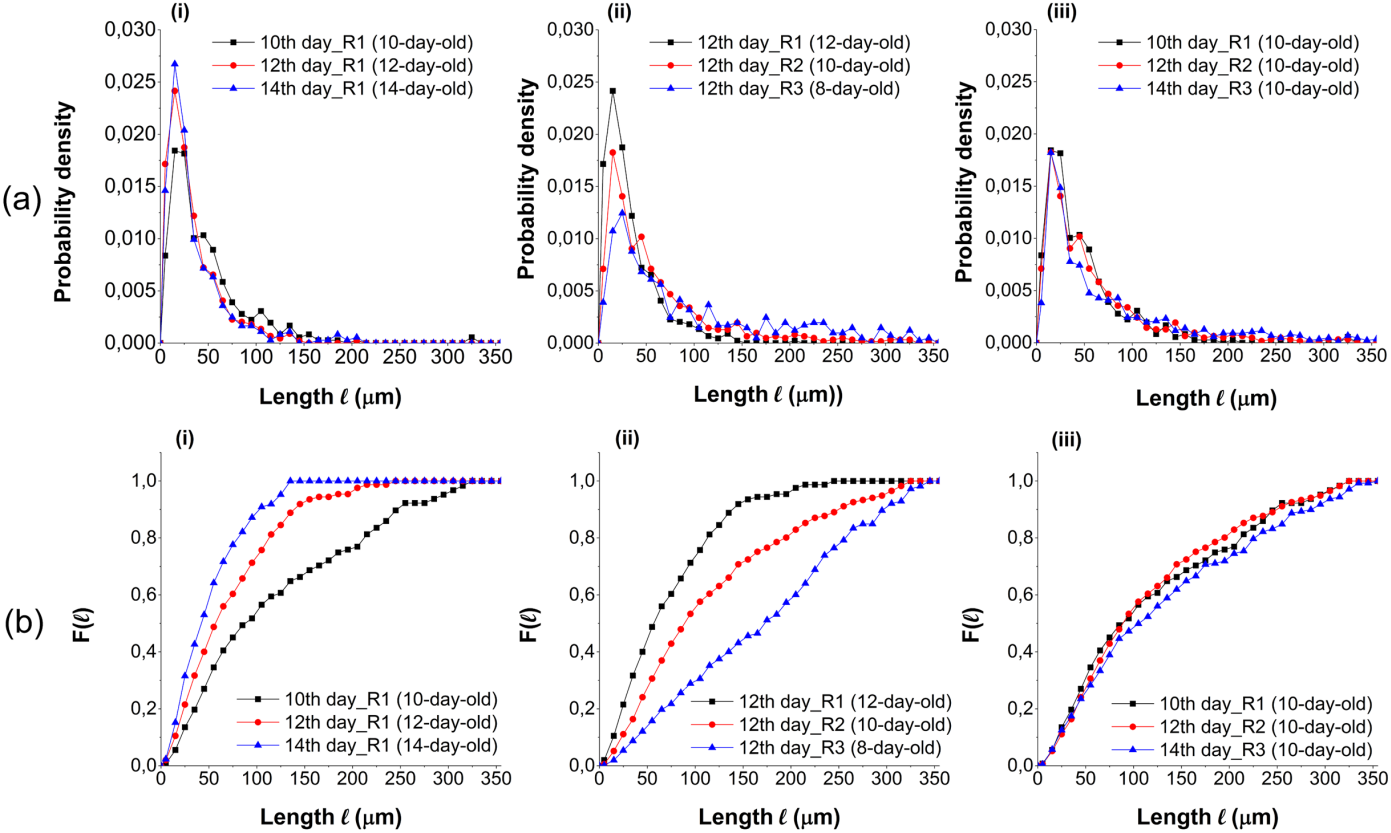
(a) Branching length distribution and (b) the corresponding weighted cumulative distribution *F*(*l*). The different graphs depict (i) the same region on different observation days, (ii) the different regions on the same observation day, (iii) the distributions of different regions on observation days selected to have the same hyphal age.

**Fig 8:**
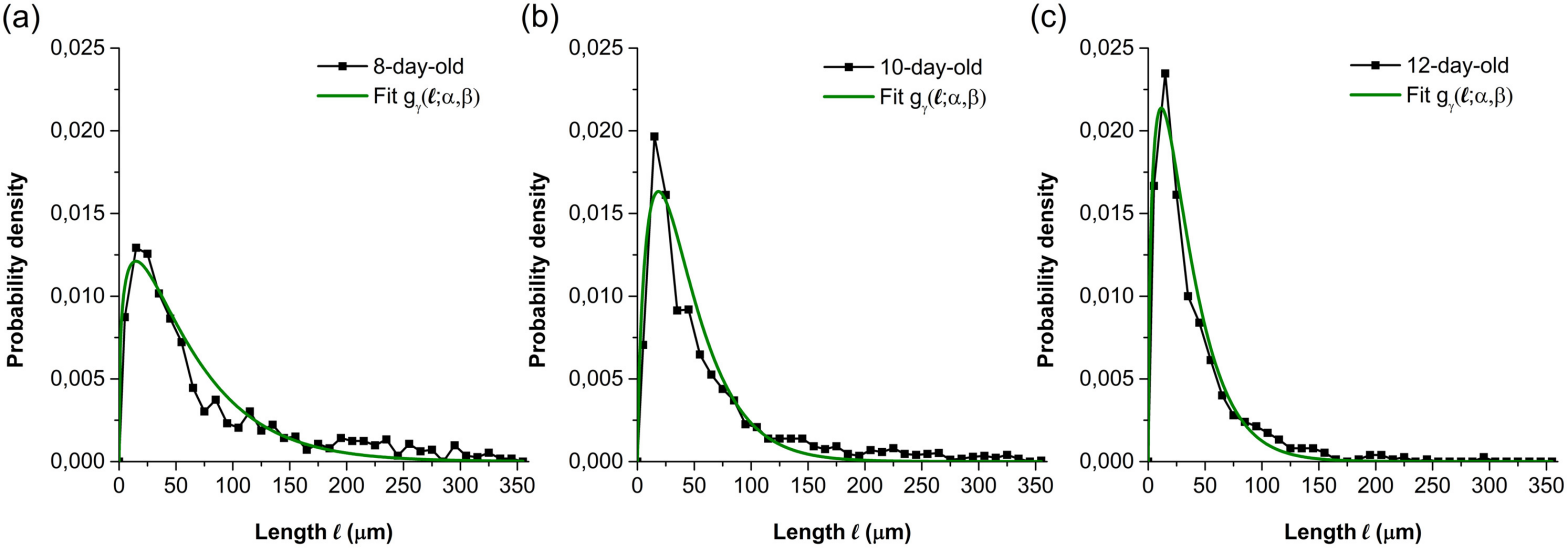
Branching length distributions at different hyphal ages and their fitted curves. (a) 8-day-old (experimental data from R2 on the 10th day and R3 on the 12th day); (b) 10-day-old (experimental data from R1 on the 10th day, R2 on the 12th day and R3 on the 14th day); (c) 12-day-old (experimental data from R1 on the 12th day and R2 on the 14th day).

In addition to branch angle, the branching length distribution, which is required to obtain the density of branches, is a complementary parameter to describe the branching pattern. The two types of branches were not distinguished while measuring the length, however, the number of LB site (*N*_*lbs*_) and AB site (*N*_*abs*_) were additionally counted. The ratio of *N*_*lbs*_/*N*_*abs*_ decreased rapidly as a function of time and subsequently leveled off and remained between 4.0 and 5.0 from the 7th day. The branching length distribution is shown in Fig 7(a) and each sample possessed a population of 300 to 500. The most frequent values were approximately 15*μm* to 25*μm*, regardless of the age of region. Their proportion increased with age of region. Indeed, as more branches appeared with time, a long branching length was divided into segments and produced several short lengths. The proportion of short lengths increased, while that of long ones decreased with time. As shown in Fig 7(a), few branching segments were longer than 200*μm*. Due to their very low probability, these long segments were not clearly visible and their evolution with age was difficult to distinguish. Yet, their importance in terms of biomass and morphology of the mycelium is important. For this reason, a weighted cumulative distribution function (Fig 7(b)) was defined to better consider the influence of the long branching segments:

$$F\left(l_{k}\right)=\frac{\sum_{i=1}^{k}f\left(l_{i}\right)l_{i}}{\sum_{i=1}^{m}f\left(l_{i}\right)l_{i}}$$ (8)

where *f* is the probability density shown in Fig 7(a); *l*_*i*_ the length of the *i*^*th*^ interval; *k* the number of the interval to be calculated and *m* the total number of the intervals. The comparison of the branching length distributions was performed among the same region (R1) of different days, among the three regions of the same day (on the 12th day) and among the regions of the same age (10-day-old). In Fig 5(b), the age of the regions on different days can be identified. Fig 7(a)-(i), 7(a)-(ii), 7(b)-(i) and 7(b)-(ii), a comparison between the regions of different ages, indicate that with the increasing of age, the proportion of short branching length arose while that of long ones reduced. It reveals that the branches emerge not only from young hyphae but also from the old parts of hyphae, and that the branch sites can be located both in the parts with low and high branch density. The coincidence of the three curves in Fig 7(a)-(iii) and 7(b)-(iii) illustrates that the branching length distributions are approximately equal at the same age of region. The error coefficient of branching length calculated by Eq (5) was shown in S4(b) Fig. Considering the 240 errors out of about 1200 branching lengths in total, there are 82 relative errors, (6.8% of 1200 measured data), greater than 5.0%. Thus, this error can be also neglected for the branching length distribution. Experimental data measured at the same age were regrouped to calculate the branching length distribution (Fig 8). The parameter identification was carried out to the branching length distributions at different hyphal ages. Fig 8 displays examples of fitted curves using a gamma distribution:

$$g_{\gamma }\left(l;\alpha ,\beta \right)=\frac{\beta^{\alpha }}{\Gamma (\alpha )}l^{\alpha -1}e^{-\beta l}$$ (9)

where *l* is the length between two adjacent branch sites; *α* and *β* are the scale and rate parameters of the gamma distribution. The low value of RSS, about 10^−5^, indicates a tight fit of the simulated data to the experimental ones. The fitted curves, which are in good agreement with the observation curves, will be used to simulate all the hyphal branching at the corresponding ages of region. The values of the scale and the rate parameter are shown in Table 2: the values of *α* are between 1.0 and 2.0 while those of *β* are less than 1.0, and increase when the branching age increases.

**Table 2:** Description and values of the identified parameters to be applied in the simulation of the mycelial growth.

Parameter	Description	Value & expression
$\bar{D_{h}}$^a^	Average diameter of hyphae	3*μm*
*k*_*active*_	Proportion of active tips	Lag phase: 0.72 Exponential phase: 0.7
*f*(*R*_*tip*_)^b^	Tip extension rate distribution	$\Gamma \left(\alpha =1.51,\beta =0.09\right)+4.65\times 10^{-6}\left(R_{tip}^{max}-R_{tip}\right)$
*k*_*lag*_	Ratio of emerged branch numbers per hypha during the lag phase to that during the exponential phase ([*φ*_*br*_]_*lag*_/[*φ*_*br*_]_*exp*_)	1/5
*f*(*θ*)	Branch angle distribution	N(*μ* = 77.6°, *σ* = 12.3°)
*N*_*lbs*_/*N*_*abs*_	Ratio of the number of LB sites to that of AB sites	4.0 − 5.0^c^
*f*(*l*)	Branching length distribution	5-day-old: Γ(*α* = 1.32, *β* = 0.022) 7-day-old: Γ(*α* = 1.71, *β* = 0.039) 9-day-old: Γ(*α* = 1.52, *β* = 0.045)

^a^ identified by the observations using CLSM;

^b^ fit to all the data from the 4th day to the 14th day (S5 Fig);

^c^ from the 7th day.

The identified parameters and their values that will be used for calibrating the model for simulating the mycelial growth are listed in Table 2. The proportion of active tips (*k*_*active*_) and the tip extension rate distribution (*f*(*R*_*tip*_)) quantify the tip extension, while the branch angle distribution (*f*(*θ*)), the ratio of *N*_*lbs*_/*N*_*abs*_ and the branching length distribution (*f*(*l*)) determine the branching pattern. In addition, the lag phase and the exponential phase are distinguished in assigning different values to *k*_*active*_ and *k*_*lag*_ in the further simulation due to the noticeable difference of hyphal growth.

## Conclusion

The live-cell imaging using CLSM described in the present work allows recording the evolution of the mycelial structure of *Postia placenta* with time. Thanks to the observations on the whole colony level, the growth characteristics can be statistically analyzed in relation with the different duration of the development of the colony. The determination of the age of different regions implies the equivalence of time and space (radius of the colony) in mycelial growth. The data analysis allows a full set of statistical expressions to be proposed to characterize the fungal growth in free growth conditions. Among interesting results, the statistical analysis shows that the hyphal growth presents certain randomness, such as the spatial distribution of tip extension rate and the selection of the branch site. Afterwards, all the quantified hyphal growth in this paper will be used to feed a 2D lattice-based model for simulating the growth of *Postia placenta*. The tip extension rate and the branch angle distribution obtained in this work will be directly used as the input parameters for the model, while the branching length distribution calculated in the simulation will be compared with the experimental one to validate the rest branching parameters. Then the calibrated model will be extended to simulate the mycelial growth in heterogeneous environment with several hypotheses, e.g. the branch angle respects its intrinsic property but the extension and activity of this emerged branch is influenced by the environmental factor. However, the observation of hyphal growth in wood is important to the further study, especially for validating a 3D model (work in progress in our team) to mimic hyphal growth in wood. So, the growth of *Postia placenta* in wood will also be observed and quantified in using nano-tomography.

## Supporting Information

S1 Fig 3D reconstruction of one section of the mycelium on the 12th day of observation. (a) Cross-sectional view of the surface and penetrative hyphae. (b) Top view of the hyphae.
(TIF)

S2 Fig 2D projection confocal images of *Postia placenta* obtained using the MIP method of the 3D images. The time evolution of the mycelial growth: the 3rd, 4th, 5th, 7th, 10th, 12th, 14th, 17th day following inoculation. The resolution is half of the original confocal images.
(TIF)

S3 Fig Tip extension during two consecutive observations. The white curve is the hypha at the 1st observation and the red curve is its shape observed 2 days later. The coordinates of the tip positions are (*x*_0_, *y*_0_) and (*x*, *y*) respectively. Δ*l*_*tip*_(*i*; *x*, *y*) is the extension length of tip *i*, i.e. the length of the path from (*x*_0_, *y*_0_) to (*x*, *y*).
(TIF)

S4 Fig Error of branch angle and branching length caused by z-direction growth of hyphae. (a) Error of branch angle, and (b) relative error of branching length calculated throughout all portions.
(TIF)

S5 Fig Fitted curve of the tip extension rate distribution. The distribution, calculated by all the data from the 4th day to the 14th day, was well fitted by a gamma distribution corrected with a linear baseline.
(TIF)
