# Correction: Morphological Characterization and Quantification of the Mycelial Growth of the Brown-Rot Fungus *Postia placenta* for Modeling Purposes

**DOI:** 10.1371/journal.pone.0189548

**Published:** 2018-05-30

**Authors:** Huan Du, Pin Lv, Mehdi Ayouz, Arnaud Besserer, Patrick Perré

There are errors in the Author Contributions. Arnaud Besserer (AB) should also be listed as an author contributing to Resources.

There is an error in the second sentence of the Abstract. The correct sentence is: The morphological characterization of *Postia placenta* was quantitatively determined, including the tip extension rate, branch angle and branching length, (hyphal length between two adjacent sites of branches and/or anastomosis).

There is an error in the second sentence under the subheading “Fluorescence staining,” in the Materials and Methods section. The correct sentence is: In each staining, the stock solution was diluted to 0.0001%(*w/v*) and was added into the culture by syringe filter (0.2*μm*).

There is an error in the seventh sentence of the last paragraph of the Materials and Methods section. The correct sentence is: Moreover, the branching length is defined as the hyphal length between two adjacent sites of branches and/or anastomosis (i.e., hyphal fusion).

There is an error in the definition of “*l*” in the seventh paragraph of the Results and Discussion section. The correct sentence should be: Fig 8 displays examples of fitted curves using a gamma distribution:

$$g_{\gamma }(l;\alpha ,\beta )=\frac{\beta^{\alpha }}{\Gamma (\alpha )}l^{\alpha -1}e^{-\beta l}$$

Where *l* is the length between two adjacent sites of branches and/or anastomosis.
