# Supplementary figures and images for: Morphological Characterization and Quantification of the Mycelial Growth of the Brown-Rot Fungus Postia placenta for Modeling Purposes
Source: PLoS One. 2016 Sep 7;11(9):e0162469. doi: 10.1371/journal.pone.0162469 (PMC5014427; doi:10.1371/journal.pone.0162469)

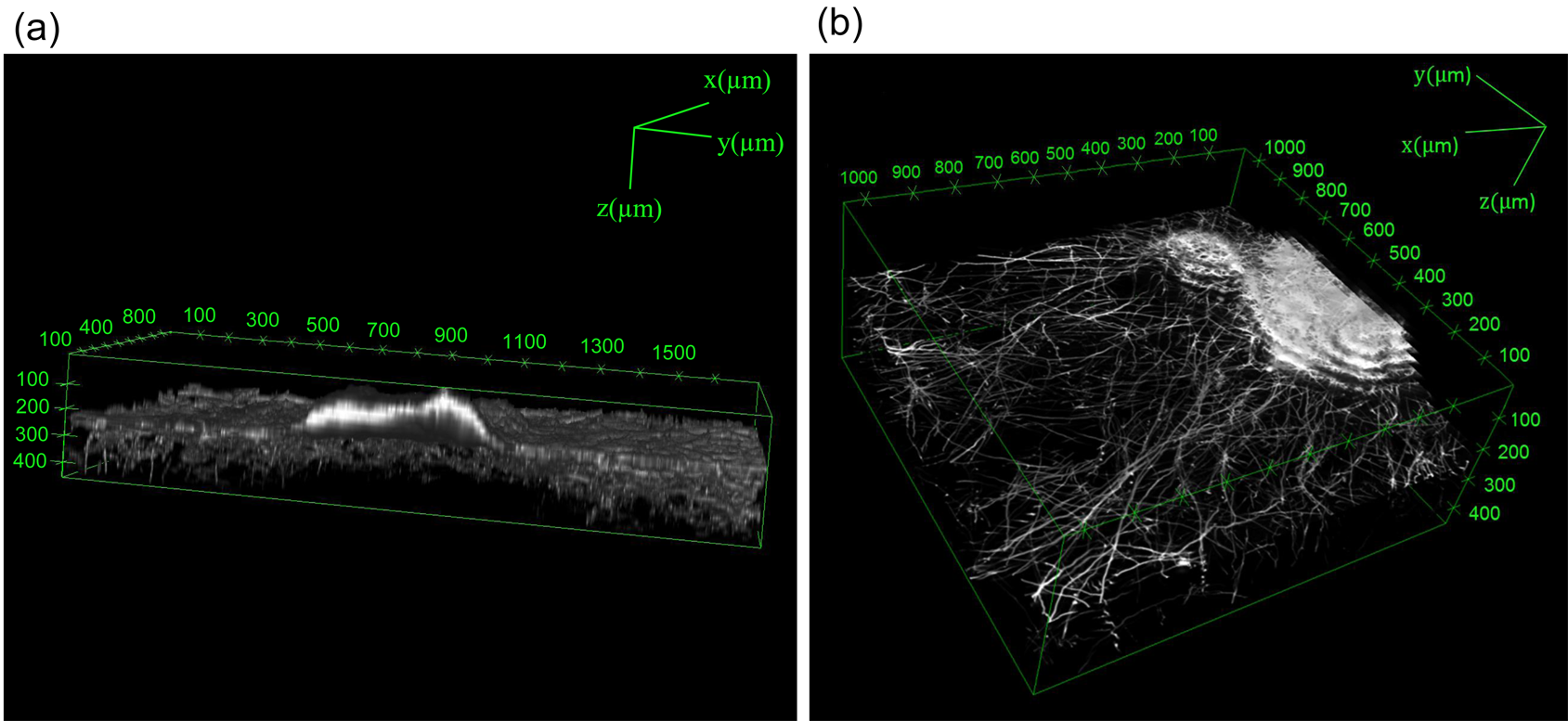

Supplement: S1 Fig — (a) Cross-sectional view of the surface and penetrative hyphae. (b) Top view of the hyphae. (TIF) [file pone.0162469.s001.tif]

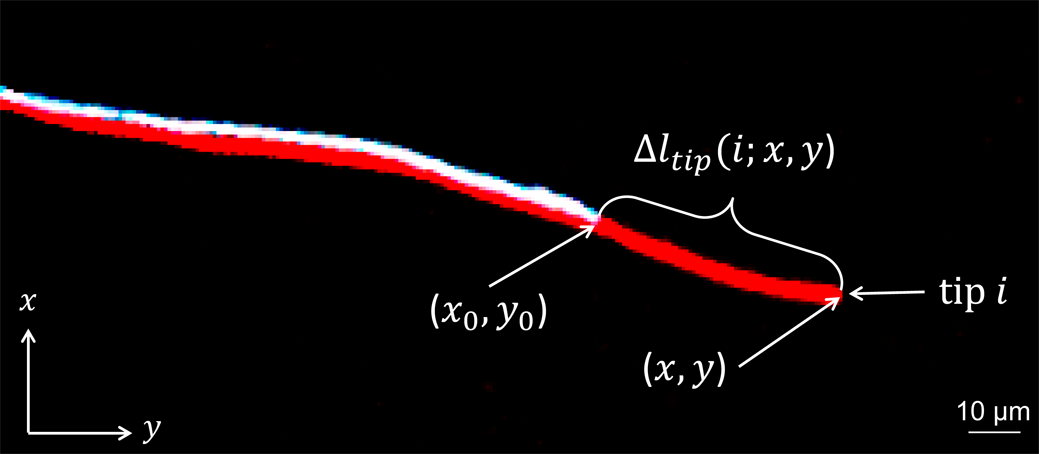

Supplement: S3 Fig — The white curve is the hypha at the 1st observation and the red curve is its shape observed 2 days later. The coordinates of the tip positions are (x0, y0) and (x, y) respectively. Δltip(i; x, y) is the extension length of tip i, i.e. the length of the path from (x0, y0) to (x, y). (TIF) [file pone.0162469.s003.TIF]

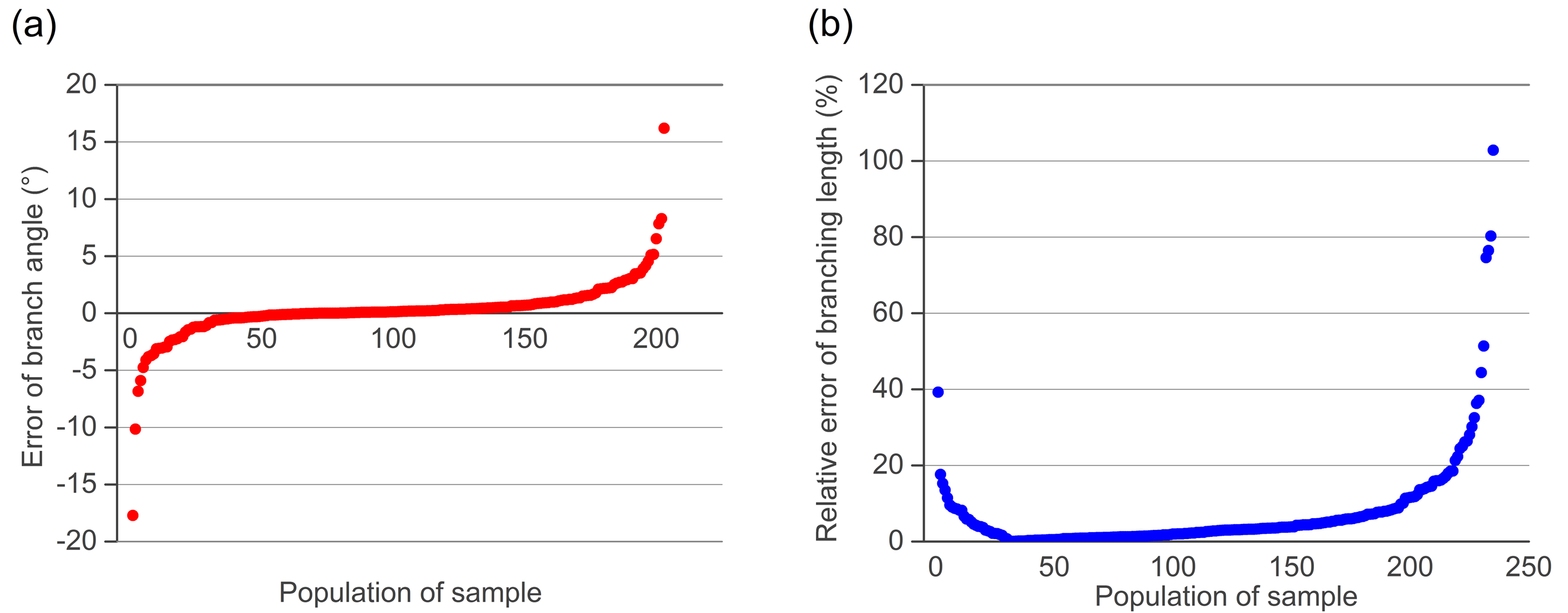

Supplement: S4 Fig — (a) Error of branch angle, and (b) relative error of branching length calculated throughout all portions. (TIF) [file pone.0162469.s004.tif]
